# Supplementary material for: Preoperative admission is non-essential in most patients receiving elective laparoscopic cholecystectomy: A cohort study
Source: PLoS One. 2023 Oct 26;18(10):e0293446. doi: 10.1371/journal.pone.0293446 (PMC10602302; doi:10.1371/journal.pone.0293446)
Supplement: S1 Table — (DOCX) [file pone.0293446.s001.docx]

**S1 Table.** Criteria for evaluating the need for preoperative treatment in the study

| Treatments | Criteria |
| --- | --- |
| Blood transfusion | Hematocrit <25% in general patients or  hematocrit <30% in patients with a risk of organ ischemia |
| Diabetes mellitus, hyperglycemic treatment | Plasma glucose >7.2 mmol/L |
| Hypertension, blood pressure controlling | Systolic blood pressure ≥160 mmHg or  diastolic blood pressure ≥100 mmHg |
| Electrolyte correction (potassium or sodium) | - Substantially below or above the normal level  - Symptomatic, such as abnormal electrocardiography  - Explainable leading etiologies such as drugs, poor intake |
| Bridging of anticoagulants | Patients requiring short-acting anticoagulants (such as heparin or low molecular weight heparin) during the surgical period, leading to interruption of their warfarin therapy |
| Preoperative cardiovascular monitoring | Unstable angina  Coronary artery disease  Arrythmias  Ventricular dysfunction  Congestive heart failure  End organ failure (e.g., end-stage renal disease, cirrhosis) |

The decision for less-frequent conditions, such as preoperative bronchodilator therapy or corticosteroid use, was made through a consensus reached between two specialist physicians (a surgeon and an anesthesiologist) during an in-person meeting, with or without subspecialist consultation.
