# Supplementary material for: Preoperative admission is non-essential in most patients receiving elective laparoscopic cholecystectomy: A cohort study
Source: PLoS One. 2023 Oct 26;18(10):e0293446. doi: 10.1371/journal.pone.0293446 (PMC10602302; doi:10.1371/journal.pone.0293446)
Supplement: S2 Table — (DOCX) [file pone.0293446.s002.docx]

**S2 Table Sensitivity analysis results for patients with symptomatic gallstones only (excluding other indications for laparoscopic cholecystectomy, n=295) and for elective laparoscopic cholecystectomy without additional procedures (excluding patients who underwent intraoperative cholangiography, n=377)**

|  | Treatment necessary^a^ | | | | | |
| --- | --- | --- | --- | --- | --- | --- |
|  | Symptomatic gallstones model (n=295) | | | No Intraoperative cholangiography model (n=377) | | |
|  | OR (95% CI) | SE | *p*-value | OR (95% CI) | SE | *p*-value |
| Age | 1.0 (1.0, 1.0) | 0.02 | 0.742 | 1.0 (1.0, 1.1) | 0.02 | 0.691 |
| ≥65 years | 1.0 (0.4, 2.7) | 0.52 | 0.951 | 1.2 (0.5, 2.7) | 0.51 | 0.664 |
| Female sex | 1.6 (0.6, 4.4) | 0.82 | 0.401 | 0.9 (0.4, 1.9) | 0.34 | 0.681 |
| BMI ≥32.5 kg/m^2^ | 1.8 (0.6, 5.6) | 1.04 | 0.296 | 2.2 (0.8, 6.3) | 1.18 | 0.151 |
| Other surgical indications^b^ |  | | | 1.1 (0.8, 1.6) | 0.20 | 0.555 |
| ASA status III | 1.1 (0.4, 3.0) | 0.56 | 0.786 | 1.2 (0.5, 2.9) | 0.53 | 0.600 |
| Comorbidity |  |  |  |  |  |  |
| One comorbidity | 10.8 (2.2, 53.2) | 8.79 | 0.003 | 8.5 (2.3, 32.4) | 5.80 | 0.002 |
| Two comorbidities | 30.9 (5.5, 174.7) | 27.33 | <0.001 | 25.1 (6.0, 105.7) | 18.41 | <0.001 |
| Three comorbidities | 105.1 (12.8, 863.5) | 112.9 | <0.001 | 118.5 (17.9, 786.2) | 114.43 | <0.001 |

^a^Treatment necessary: preoperative treatment that was documented in the records, excluding cases of over-treatment and including cases of ­­under-treatment after exclusion.

^b^Other surgical indications: indications for laparoscopic cholecystectomy other than symptomatic gallstones.

ASA: American Society of Anesthesiologists, BMI: body mass index, CI: confidence interval, OR: odds ratio, SE: standard error.
