# Supplementary material for: Preoperative admission is non-essential in most patients receiving elective laparoscopic cholecystectomy: A cohort study
Source: PLoS One. 2023 Oct 26;18(10):e0293446. doi: 10.1371/journal.pone.0293446 (PMC10602302; doi:10.1371/journal.pone.0293446)
Supplement: S1 File — (PDF) [file pone.0293446.s003.pdf]

STROBE Statement—Checklist of items that should be included in reports of ***cohort studies***
Study's title: Preoperative admission is non-essential in most patients receiving elective laparoscopic cholecystectomy: a cohort study

	Item No	Recommendation
**Title and abstract**	1	(☑) Indicate the study's design with a commonly used term in the title or the abstract
		(☑) Provide in the abstract an informative and balanced summary of what was done and what was found
**Introduction**		
Background/rationale	2	☑ Explain the scientific background and rationale for the investigation being reported
Objectives	3	☑ State specific objectives, including any prespecified hypotheses
**Methods**		
Study design	4	☑ Present key elements of study design early in the paper
Setting	5	☑ Describe the setting, locations, and relevant dates, including periods of recruitment, exposure, follow-up, and data collection
Participants	6	(☑) Give the eligibility criteria, and the sources and methods of selection of participants. Describe methods of follow-up
		(None) For matched studies, give matching criteria and number of exposed and unexposed
Variables	7	☑ Clearly define all outcomes, exposures, predictors, potential confounders, and effect modifiers. Give diagnostic criteria, if applicable
Data sources/ measurement	8*	☑ For each variable of interest, give sources of data and details of methods of assessment (measurement). Describe comparability of assessment methods if there is more than one group
Bias	9	☑ Describe any efforts to address potential sources of bias
Study size	10	☑ Explain how the study size was arrived at
Quantitative variables	11	☑ Explain how quantitative variables were handled in the analyses. If applicable, describe which groupings were chosen and why
Statistical methods	12	(☑) Describe all statistical methods, including those used to control for confounding
		(☑) Describe any methods used to examine subgroups and interactions
		(☑ No missing data) Explain how missing data were addressed
		(☑) If applicable, explain how loss to follow-up was addressed
		(None) Describe any sensitivity analyses
**Results**		
Participants	13*	(☑) Report numbers of individuals at each stage of study—eg numbers potentially eligible, examined for eligibility, confirmed eligible, included in the study, completing follow-up, and analysed
		(NA) Give reasons for non-participation at each stage
		(☑) Consider use of a flow diagram
Descriptive data	14*	(☑) Give characteristics of study participants (eg demographic, clinical, social) and information on exposures and potential confounders
		(☑) Indicate number of participants with missing data for each variable of interest
		(☑) Summarise follow-up time (eg, average and total amount)
Outcome data	15*	(☑) Report numbers of outcome events or summary measures over time
Main results	16	(☑) Give unadjusted estimates and, if applicable, confounder-adjusted estimates and

		their precision (eg, 95% confidence interval). Make clear which confounders were adjusted for and why they were included
		(☑) Report category boundaries when continuous variables were categorized
		(☐) If relevant, consider translating estimates of relative risk into absolute risk for a meaningful time period
Other analyses	17	Report other analyses done—eg analyses of subgroups and interactions, and sensitivity analyses
**Discussion**		
Key results	18	☑ Summarise key results with reference to study objectives
Limitations	19	☑ Discuss limitations of the study, taking into account sources of potential bias or imprecision. Discuss both direction and magnitude of any potential bias
Interpretation	20	☑ Give a cautious overall interpretation of results considering objectives, limitations, multiplicity of analyses, results from similar studies, and other relevant evidence
Generalisability	21	☑ Discuss the generalisability (external validity) of the study results
**Other information**		
Funding	22	☑ Give the source of funding and the role of the funders for the present study and, if applicable, for the original study on which the present article is based

*Give information separately for exposed and unexposed groups.

Note: An Explanation and Elaboration article discusses each checklist item and gives methodological background and published examples of transparent reporting. The STROBE checklist is best used in conjunction with this article (freely available on the Web sites of PLoS Medicine at http://www.plosmedicine.org/, Annals of Internal Medicine at http://www.annals.org/, and Epidemiology at http://www.epidem.com/). Information on the STROBE Initiative is available at http://www.strobe-statement.org.
