# Supplementary material for: Preoperative admission is non-essential in most patients receiving elective laparoscopic cholecystectomy: A cohort study
Source: PLoS One. 2023 Oct 26;18(10):e0293446. doi: 10.1371/journal.pone.0293446 (PMC10602302; doi:10.1371/journal.pone.0293446)
Supplement: Attachment — Submitted filename: Response to reviewer.docx [file pone.0293446.s005.docx]

Dr. Fabrizio D'Acapito

Academic Editor

Dr. Emily Chenette

Editor-in-Chief

*PLOS ONE*

*September 22, 2023*

Dear Dr. Fabrizio D'Acapito and Dr. Emily Chenette

Re: Manuscript reference No.: ONE-D-23-13647R1

Please find attached a second revised version of our manuscript " Preoperative admission is non-essential in most patients receiving elective laparoscopic cholecystectomy: a cohort study.", which we would like to resubmit for publication as a Research article in *the PLOS ONE*.

The comments of the editor and reviewer were highly insightful and enabled us to greatly improve the quality of our manuscript. In the following pages are our point-by-point responses to each of the comments of the reviewers.

Revisions in the text are shown using red highlight for additions, and strikethrough font *[~~example~~]* for deletions. All response's page and lines number are corresponding to the 'Revised Manuscript with Track Changes' file. We hope that the revisions in the manuscript and our accompanying responses will be sufficient to make our manuscript suitable for publication *the PLOS ONE*.

We shall look forward to hearing from you at your earliest convenience.

Yours sincerely,

Suppadech Tunruttanakul

Department of Surgery, Sawanpracharak Hospital, 43 Atthakawee Road, Muang, Nakhon Sawan, 60000, Thailand

Tel: +66 897590770

Fax: +6656 219899

Email: [sdtaaa@yahoo.com](mailto:sdtaaa@yahoo.com); [suppadech_t@spr.go.th](mailto:suppadech_t@spr.go.th)

**Responses to the Editor Comments**

We thank again to to Dr. Fabrizio D'Acapito for providing us with detailed and valuable comments. Your valuable input has been instrumental in improving our research. We have taken each comment into careful consideration and made the necessary revisions accordingly. Here is a summary of how we addressed each comment: (the specified page and line are according to the “Revised manuscript with Track Changes” file)

**Comment 1.** I thank the authors for their effort in making the suggested changes to their study.
The result is good, but in agreement with the reviewer 3 I think some further minor adjustments are needed (Beginning with point 2 and 3 proposed by reviewer 3).
Since the aim of the paper is to change, in a precise territorial area, the current attitude regarding the treatment of gallbladder lithiasis and to increase the use/optimization of day surgery, it is imperative that the definitions and indications for treatments are clear and precise, as well as the criteria to perform preoperative investigations must be well defined.

***Response****:* We appreciate the editor's feedback. In this updated version, we have attempted to incorporate your and reviewer 3's suggestions. We hope that these revisions adequately address the comment.

**Comment 2.** As the reviewer 3 also suggested, it is necessary to better clarify (probably a translation problem) the concept of "required treatment" and "actual treatment rendered."

***Response****:* We appreciate the editor's comment. Upon careful review, we fully acknowledge and agree with your and reviewer 3's concerns. Consequently, we sought guidance from our language editor, resulting in the following changes: we replaced "required treatment" with "treatment necessary," and "actual treatment rendered" with "treatment received." These modifications have been consistently applied throughout the text, and we have included additional clarifying messages as follow:

The definitions of the terms have been updated from page 8, line 5 to page 9, line 6, as: “The primary objective of this study was to evaluate the treatment and monitoring received by patients during their preoperative admission, excluding care provided on the day of surgery. Two participating specialists, a surgeon and an anesthesiologist, separately reviewed the patient data and later held an in-person meeting to review their findings. In cases of inconclusive results, a subspecialist in internal medicine was consulted. The criteria for necessary preoperative treatment are outlined in S1 Table. Based on the described process, preoperative treatment was classified into three categories: “treatment received”, over-treatment, and under-treatment. Subsequently, the category of “treatment necessary”, representing necessary treatment for patients, was determined based on the aforementioned categories. The details of each category are as follows:

- “Treatment received” referred to treatments that were provided and documented in the patient's records.

- Over-treatment denoted treatment that should not have been given.

- Under-treatment referred to treatment that was necessary but not given.

- “Treatment necessary” referred to “treatment received”, excluding cases of over-treatment and including cases of under-treatment after exclusion.

In our analysis, each patient was treated as an individual unit for evaluation. Including under-treatment after excluding over-treatment was intended to prevent the exclusion of patients who experienced both under-treatment and over-treatment. "Treatment necessary" comprised all patients who received the preoperative treatment (i.e., the "treatment received" group), as well as those who required treatment but did not receive it or received insufficient treatment (i.e., the under-treatment group). However, "treatment necessary" excluded patients in the over-treatment group.”

**Comment 3.** Regarding reference item 31 (in the discussion) is misplaced , in fact “The chapter focuses on perioperative assessment and anesthetic considerations of the trauma patient specially focused on difficult airway rescue and management by the various intubation techniques, precautions during extubation, and postoperative care “
Bibliographic entry 32 is also very irrelevant, in fact it refers to 58 patients undergoing general surgery out of 499 examined who did not undergo pre-operative testing. Nor is the procedure performed specified.

***Response****:* We sincerely appreciate the editor's insightful comments and apologize for any oversight in reference selection. We have thoroughly reevaluated the references and incorporated more pertinent ones. Specifically, we have updated the passage to (page 19, line 16 to 20) "Recent guidelines from the United Kingdom (National Institute for Health and Care Excellence) have shifted away from recommending routine preoperative tests [32]. Moreover, these tests (if done routinely) have the potential to cause harm and incur unnecessary costs without significantly altering preoperative management [33]. "

The replaced references are as follows:

32.	National Guideline Centre (UK). Preoperative Tests (Update): Routine Preoperative Tests for Elective Surgery. London: National Institute for Health and Care Excellence (NICE); 2016 Apr. (NICE Guideline, No. 45.) 1, Guideline summary.

33.	Martin SK, Cifu AS. Routine Preoperative Laboratory Tests for Elective Surgery. JAMA. 2017;318(6):567-8.

**Group of relevant comments**

Complications according to which classification were classified (Clavie-Dindo? Other?).
I also request an extensive linguistic revision of the text, which is difficult to understand in some passages.
I ask that in the introduction the first sentence (defining gallbladder lithiasis) and in lines 3-4 the definition of cholecystectomy (which should be known to all surgeons) be removed.
In the materials and methods page 5 lines 24-27 the choice of cut off for age is not clearly defined.
I read reference 14 in lancet but did not find the grouping you propose. Is yours an adaptation from the original?
Page 7 lines 1-5 it is necessary to define the indication to IOC.
Page 7 line 25 : define "complete blood count"
Page 7 line 26: Why EKG only to those over 45? According to what guidelines was this choice made?
P8 lines 1-2. According to which guidelines is it correct to submit a patient who has not had liver function tests to cholecystectomy? Some tests may raise suspicion of choledochal lithiasis or the presence of other pathologies that may not be immediately understood postoperatively.
Page 8 lines 19-26 and page 9 lines 1-5 the ideas expressed need to be rewritten more clearly.
What does the one-month follow-up program include: an outpatient visit? The execution of blood tests? An ultrasound examination? Anything else?
In the results section, page 11 line 30. The 798 cancellations refer to what type of procedures? If they are not cholecystectomies why provide this figure?
What is meant by "cardiovascular instability monitoring"?
**Response**: We sincerely appreciate the editor's meticulous review and valuable comments. Your thoughtful evaluation significantly improved our manuscript. As these comments revolve around enhancing clarity of expression, we will collectively address them for better coherence.

**1.** I ask that in the introduction the first sentence (defining gallbladder lithiasis) and in lines 3-4 the definition of cholecystectomy (which should be known to all surgeons) be removed.
**Response:** We have eliminated the initial paragraph in the introduction and inserted the sentence “Laparoscopic cholecystectomy (LC), the surgical removal of the gallbladder, is a frequently performed procedure [1] used to treat various complications related to gallstones [2].” at the beginning of the second paragraph, which is now the first paragraph in the revised version (page 4, line 9 to 10).

**2.** In the materials and methods page 5 lines 24-27 the choice of cut off for age is not clearly defined.
**Response:** We apologize for the previous confusion regarding the cut-off. We have revised the sentence as follows (page 5, line 25 to page 6, line 1): “The patient's age was recorded in years and grouped into two categories: below 65 years old and 65 years old or older (the cutoff in the review by Orimo et al. [11]).” Additionally, we have updated the reference to: “Orimo H, Ito H, Suzuki T, Araki A, Hosoi T, Sawabe M. Reviewing the definition of 'elderly'. Geriatrics & Gerontology International. 2006;6(3):149-58.”

**3.** I read reference 14 in lancet but did not find the grouping you propose. Is yours an adaptation from the original?
**Response:** We sincerely apologize for not thoroughly checking this criterion. We highly appreciate the editor's diligence in pointing out this oversight. The cutoffs have been revised to <27.5, 27.5 to <32.5, and ≥32.5 kg/m2. The terms "class I" and "class II" obesity were removed, given the lack of consensus on cutoffs in Asian countries, including the nomenclature for these criteria. These changes have been implemented throughout the entire manuscript. Furthermore, we have recalculated and revised the results in the text and tables. Some sentences were removed accordingly. Below is a detailed list of the changes made:

1). Page 6, line 1 to 3 in Material and Methods: “BMI was recorded in kg/m^2^ and divided into three categories based on the Asian obesity cutoff value for public health action: <27.5 kg/m^2^, 27.5 to <32.5, and ≥32.5 kg/m^2^ [12].”

2). Page 12, line 6 to 9 in Results: “Most patients were women (77.0%); 25.7% (104 patients) of the total patients were obese with BMI 27.5 to <32.5 kg/m2, and 9.4% (38 patients) were BMI ≥32.5.”

3). Page 14, line 18 to 19 in Results: Patients' age, “BMI greater than 32.5 kg/m^2^, presence of IOC, surgical indications, and ASA status were not associated with preoperative treatments.”

4). Page 15, line 6 to 9 in Results. The sentence “With our sample size, no variable was found to significantly predict medical complications, although class II obesity trended toward requiring both preoperative treatment and increased medical complications (odds ratio and p-value of 2.9 and 0.222, and 9.8 and 0.090, respectively).” has been revised to “With our sample size, no variable was found to significantly predict medical complications.”

5). In the Discussion, two messages regarding incorrect classifications and their associated results have been removed as follows:

- Page 18, line 22 to 25: “Class II obesity showed a trend toward higher treatment requirements and medical complications, with an odds ratio of 2.9 (*p*-value 0.222) and 9.8 (*p*-value 0.090), respectively. The limited number of class II obese patients (n=10) may have contributed to the non-significance of the results.” has been removed.

- Page 21, line 8 to 10: “It is possible that the incidence of OSA was low, given the small proportion of obese patients (9.4%, or 38 patients with class I and II obesity) who also have a high likelihood of OSA [39].” has been removed.

This is because body mass index ≥32.5 kg/m2 no longer trends towards both treatment requirements and the occurrence of medical complications. Our incidence of obesity, considering the revised cut-off, is also substantial, up to 35.1% (body mass index ≥27.5 kg/m2)."

6) In Tables 1, 2, 3, and S1: All criteria have been revised accordingly, necessitating a comprehensive re-calculation and adjustment of all results. Consequently, numerical results must be revised throughout the manuscript, including the abstract. Please look for red highlights for additions and use strikethrough font for deletions."

**4.** Page 7 lines 1-5 it is necessary to define the indication to IOC.

**Response:** We appreciate the editor's feedback. We have provided additional details regarding the indications for intraoperative cholangiography (IOC). The updated statement is as follows (page 6, line 21 to page 7, line 2): “Regarding the presence of IOC parameters, in our department, selective IOC was indicated for patients with symptomatic gallstones who had a relatively low but non-negligible risk of choledocholithiasis. This included patients with a clinical history of jaundice, pancreatitis, abnormal liver function test results, bile duct dilatation detected in imaging studies, or the patients at intermediate risk for choledocholithiasis according to guidelines [14]. In cases with a greater suspicion of choledocholithiasis (high-risk for choledocholithiasis, which includes clinical cholangitis or choledocholithiasis detected in imaging studies), alternative modalities such as endoscopic retrograde cholangiography were used [14].” The reference has been changed from 'ASGE guidelines' to 'ESGE guidelines' as it is more coherent with our protocol.

**5.** Page 7 line 25: define "complete blood count"

**Response:** We appreciate the editor's comment. The phrasing has been updated to (page 7, line 21 to 23): “Chest radiography and a complete blood count (blood test that evaluates the total numbers and characteristics of cell components in a patient's blood, including red and white blood cells and platelets) were performed for all patients.”

**6.** Page 7 line 26: Why EKG only to those over 45? According to what guidelines was this choice made?

**Response:** We appreciate the editor's feedback and apologize for any confusion. The criteria details have been updated to specify (page 7, line 25 to page 8, line 1): “Our hospital's preoperative screening test protocol with respect to patients’ age (i.e., blood tests, chest radiography, and electrocardiogram testing) has been adopted from the practice advisory for pre-anesthesia evaluation by the Royal College of Anesthesiologists of Thailand [17].”

The reference 'No 17' pertaining to the guideline has been supplemented with the relevant information: “17. The Royal College of Anesthesiologists of Thailand. Practice advisory for preanesthesia evaluation. 2019. Available from: <https://www.rcat.org/_files/ugd/82246c_6386a015c1574075a50dff87cfc2b060.pdf>”

**7.** P8 lines 1-2. According to which guidelines is it correct to submit a patient who has not had liver function tests to cholecystectomy? Some tests may raise suspicion of choledochal lithiasis or the presence of other pathologies that may not be immediately understood postoperatively.

**Response:** We thank the editor for this insightful comment, and we deeply regret the oversight. Your feedback is highly valuable. We have revised the message as follows (page 8, line 1 to 4): “Additional laboratory tests were organized based on specific comorbidities, such as blood sugar testing for diabetes mellitus. Liver function tests were conducted for all patients undergoing LC.”

8. Page 8 lines 19-26 and page 9 lines 1-5 the ideas expressed need to be rewritten more clearly.

What does the one-month follow-up program include: an outpatient visit? The execution of blood tests? An ultrasound examination? Anything else?

Complications according to which classification were classified (Clavie-Dindo? Other?).

**Response:** We express our sincere gratitude to the editor for providing these valuable comments. Since they fall within the same context, we will address them collectively.

The classification of complications was updated according to the Clavien-Dindo criteria, as per your suggestion. We have included this information in the Material and Methods section, along with the relevant reference (page 9, line 21 to 26): “Complications were classified according to Clavien–Dindo classification. In brief, Grade I indicates any deviation from the normal postoperative course not requiring treatment; Grade II involves the need for pharmacological treatment; Grade III requires surgical, endoscopic, or radiological intervention; Grade IV denotes life-threatening complications, and Grade V signifies patient death [20].” Additionally, we have revised the discussion of complications in the Results section (page 14, line 20 to page 15, line 6): “Complications occurred in 17 (4.2%) patients, with no mortality; 58.8% of complications were surgery-related (10 patients, 2.8%), including two cases of major morbidity (grade III, bile duct injury requiring surgical reconstruction). Medical complications were experienced by nine patients (2.2%), with two severe cases (grade IV, 0.5%) including one cerebrovascular infarction and one readmission for lobar pneumonia with respiratory failure. Five patients had both grade II surgical and medical complications. Three patients (0.7%) experienced retained common bile duct stones.”

We have included additional details about the follow-up program in the Material and Methods section as follows (page 10, line 10 to 13): *“*Our surgical department routinely conducts a 1-month follow-up. Assessment of clinical symptoms, physical examinations, and reviews of histologic reports are performed at outpatient visits during follow-up for patients undergoing LC. Additionally, liver function tests are used to monitor patients undergoing LC and IOC.”

The unclear messages have been re-edited for language as per your suggestion. The modifications now span from page 9, line 20 to page 10, line 16.

**9.** In the results section, page 11 line 30. The 798 cancellations refer to what type of procedures? If they are not cholecystectomies why provide this figure?

**Response:** We appreciate the editor's feedback and apologize for including unrelated details. Following your suggestion, the unrelated message has been removed, as shown (page 12, line 12 to 14): “Surgery cancellations related to the study protocol were observed in two cases (0.5%).”

**10.** What is meant by "cardiovascular instability monitoring"?

**Response:** We thank the editor again for this comment. The detail regarding "cardiovascular instability monitoring" has been added in the Material and Method (page 9, line 9 to19) as: “All necessary preoperative cardiovascular instability monitoring was also considered preoperative treatment (S1 Table). Monitoring preoperative cardiovascular instability is a standard protocol at our hospital for high-risk non-cardiovascular surgical patients. On the preoperative day, anesthesiologists require an extended monitoring period, which includes non-invasive blood pressure and vital sign measurement, continuous electrocardiographic monitoring, and peripheral pulse oximetry measurements [19]. Additionally, in-patient preoperative anesthesiology visits, checklists, and a review of PAC assessments are conducted. Additional monitoring during the operative and postoperative periods is also implemented but beyond the scope of this study. This process is designed to enhance the safety of surgery for patients deemed at high risk for perioperative complications (S1 Table).” The relating reference has been added (19. Aseni P, Orsenigo S, Storti E, Pulici M, Arlati S. Current concepts of perioperative monitoring in high-risk surgical patients: a review. Patient Saf Surg. 2019;13:32.)

**Editing by Authors in Addition to Editor or Reviewer Comments**

In the abstract (page 2, line 20 to 21), the sentence “The cohort had 4.2% morbidity (2.2% severe medical complications), with no mortality.” has been revised to “The cohort had 4.2% morbidity (2.2% medical complications), with no mortality.” by removing 'severe' from medical complications, as the revised version is more accurate regarding the study results."

**Responses to the comments of Reviewer #3**

We express our gratitude to Reviewer #3 for their detailed and invaluable feedback. Your constructive comments have significantly contributed to enhancing the rigor of our study. We carefully considered each comment and made the necessary revisions accordingly. Here is a summary of the actions taken to address your valuable input: (the specified page and line are according to the “Revised manuscript with Track Changes” file)

**1.** One additional Limitation that should be mentioned in the Discussion is that the people who rated treatments as necessary or unnecessary were aware of the study hypotheses, so they may have been biased in their ratings.

**Response:** We appreciate Reviewer #3 for highlighting this concern, and we completely agree. In response to your suggestion on page 20, line 26 to page 21, line 2, we have included a limitation as follows: “In connection with the second limitation, two participating specialists who assessed the primary outcome were aware of the study's hypotheses. Consequently, there is a possibility of bias in their assessments of whether preoperative treatment was necessary.”

**2.** This may be more an issue with the translation than the underlying concepts being used, but I still have a problem understanding the grouping and the coding of the primary outcome variable. This sentence does not make sense to me: “none of the patients with ASA status I received both actual treatment rendered and the required treatment. “I don’t understand how a patient could have not received the actual treatment rendered: if it was actual and rendered, then it must have been received. What am I missing? The entire definition of the outcome variable is similar: the words do not make sense.

**Response:** We appreciate Reviewer #3 for pointing out this concern and apologize for any remaining confusion. We have revised the sentence (page 13, line 22 to 24) as follows: “Notably, in our dataset, patients with ASA status I did not receive preoperative treatment ('treatment received') or need necessary treatment (“treatment necessary”) on the preoperative admission date.”

To address the confusion surrounding the concepts of “actual treatment rendered” and “required treatment,” we consulted with our language editor and made the following changes: we replaced “required treatment” with “treatment necessary,” and “actual treatment rendered” with “treatment received.” These modifications have been applied consistently throughout the text. Additionally, to provide further clarity, we have included explanatory statements as (page 8, line 5 to page 9, line 6):

“The primary objective of this study was to evaluate the treatment and monitoring received by patients during their preoperative admission, excluding care provided on the day of surgery. Two participating specialists, a surgeon and an anesthesiologist, separately reviewed the patient data and later held an in-person meeting to review their findings. In cases of inconclusive results, a subspecialist in internal medicine was consulted. The criteria for necessary preoperative treatment are outlined in S1 Table. Based on the described process, preoperative treatment was classified into three categories: “treatment received”, over-treatment, and under-treatment. Subsequently, the category of “treatment necessary”, representing necessary treatment for patients, was determined based on the aforementioned categories. The details of each category are as follows:

- “Treatment received” referred to treatments that were provided and documented in the patient's records.

- Over-treatment denoted treatment that should not have been given.

- Under-treatment referred to treatment that was necessary but not given.

- “Treatment necessary” referred to “treatment received”, excluding cases of over-treatment and including cases of under-treatment after exclusion.

In our analysis, each patient was treated as an individual unit for evaluation. Including under-treatment after excluding over-treatment was intended to prevent the exclusion of patients who experienced both under-treatment and over-treatment. "Treatment necessary" comprised all patients who received the preoperative treatment (i.e., the "treatment received" group), as well as those who required treatment but did not receive it or received insufficient treatment (i.e., the under-treatment group). However, "treatment necessary" excluded patients in the over-treatment group.”

**3.** This paragraph is confusing: “A goodness of fit test was conducted for two logistic regression models: the required treatment model and the medical complication model. The required treatment model had a p-value of 0.810 0.609, and the medical complication model had a p-value of 0.181 0.333, and the prolonged hospital stays model had a p-value of 0.150. These results suggest that both all models were a good fit for the data, as the numbers of occurrences were not significantly different from those predicted by the models [13] [21]” Akaike’s Information Criterion (AIC) is often used to evaluate goodness of fit to the data, but it is generally compared between nested models without using a p value. It is not clear what kind of ‘goodness of fit’ test was used, why it produces several p values per model, and what a non-significant p value for this test means. Perhaps they are referring to model calibration: the agreement between predicted and observed incidence of the outcome across a range of predicted probabilities, often grouped into deciles. This is sometimes tested using the Hosmer-Lemeshow test, where a non-significant finding would mean good calibration. If that is true, please state this.

**Response:** We greatly appreciate Reviewer #3 for this insightful comment. Your suggestions are consistently clear and accurate. In the previous version of the manuscript, we calibrated our models using the Pearson chi-square test. However, as you correctly pointed out, the Hosmer-Lemeshow test would be more appropriate. In response to this comment, we have made the following revisions to our manuscript:

1. In the Material and Methods section (page 11, line 3 to 5), the text has been revised to: “The goodness of fit of the logistic regression models was tested using the Hosmer–Lemeshow test. This assessment involves grouping the observations into deciles (groups of 10) based on their predicted probabilities [23].”

2. In the Results section (page 15, line 14 to 22), we have recalculated the results and revised the text to: “A goodness of fit test (model calibration) was conducted for logistic regression models, assessing the agreement between predicted values from the model and observed values. A non-significant result was interpreted as indicative of good calibration [23]. Regarding models in Table 3, the treatment necessary model had a *p*-value of 0.221, the medical complication model had a *p*-value of 0.138, and the prolonged LOHS model had a *p*-value of 0.126. These results suggest that all models had a proper ability to describe the response variable.”
